# Baseline electrolyte disorders predict disease severity and mortality in patients with COVID-19

**DOI:** 10.1097/MD.0000000000032397

**Published:** 2022-12-23

**Authors:** Nevin Taci Hoca, Bahadir M. Berktaş

**Affiliations:** a Department of Pulmonology, Faculty of Medicine, Gazi University, Emniyet Mah, Yenimahalle, Ankara, Turkey; b Department of Pulmonology, Faculty of Medicine, Health Sciences University, Atatürk Sanatorium Training and Research Hospital, Sanatorium Cad, Keçiören, Ankara, Turkey.

**Keywords:** COVID-19, electrolyte disorders, hypocalcemia, hyponatremia, hypopotassemia

## Abstract

Distinguishing critical laboratory biomarkers for disease severity at the time of hospital presentation is important for early identification of patients who are most likely to have poor outcomes and effective use of health resources. This study aimed to evaluate whether electrolyte imbalances on hospital admission predict severe disease and mortality in patients with coronavirus disease 2019 (COVID-19). We retrospectively collected data on the blood electrolyte concentrations of 286 COVID-19 patients at admission. The correlations between electrolyte imbalances, inflammation, and thrombosis markers in COVID-19 patients were also evaluated. We assessed the predictive performance of baseline blood electrolyte concentrations for severe disease and death using receiver operating characteristic curve analysis and multivariate logistic regression methods. Abnormalities in serum sodium, calcium, and potassium levels at admission were found at 20.6%, 14%, and 4.2%, respectively in this study. In the receiver operating characteristic curve analyses, hypocalcemia and hyponatremia effectively predicted disease progression to hospitalization (area under the curve 0.82, *P* < .001 and 0.81, *P* < .001, respectively) and 30-day mortality (area under the curve 0.85, *P* < .001 and 0.91, *P* < .001, respectively). In the multivariate logistic regression analysis, baseline hypocalcemia was identified as an independent risk factor associated with the risk of hospitalization (*β* = 2.019, *P* = .01; odds ratio: 7.53). Baseline hypocalcemia and hyponatremia effectively predicted disease progression toward hospitalization and 30-day mortality in patients with COVID-19. Clinicians should closely follow up or reevaluate COVID-19 patients with baseline electrolyte disorders.

## 1. Introduction

As of September 25, 2022, the number of confirmed coronavirus disease 2019 (COVID-19) cases reached over 612 million, and 6.5 million patients died from this disease globally.^[1]^ Many populations remain vulnerable owing to limited access to vaccines, especially in low-income countries. The Omicron variant can cause severe illness and death in unvaccinated individuals and in high-risk groups. These patients may also serve as reservoirs of transmission. The immunity provided by vaccinations and natural infections may diminish over time, and there is no guarantee that vaccines will work against future variants. The sustained management of COVID-19 will continue for a long time. All information provided by studies on COVID-19 is needed to prepare for the next pandemic.^[2]^

The multisystem nature of COVID-19 has been well-demonstrated.^[3]^ Angiotensin converting enzyme-2 receptor is intensely expressed in the kidney tissue, so severe acute respiratory syndrome coronavirus 2 (SARS-CoV-2) can cause kidney injury.^[4]^ SARS-CoV-2 can impair the normal intestinal mucosa and the normal intestinal flora.^[5]^ COVID-19’s effects on the kidneys and gastrointestinal tract can cause an imbalance of fluid and electrolytes owing to the essential role of these organs in the fluid and electrolyte balance in the body.^[6]^ Pro-inflammatory cytokines can provoke hypothalamic arginine vasopressin secretion.^[7]^ IL-6 levels were found to be inversely proportional to serum sodium levels in SARS-COV2 patients.^[8]^ In 4664 patients hospitalized with COVID-19, hyponatremia and hypernatremia at hospitalization were reported in 20% and 3.7%, respectively.^[9]^ The first severely hypocalcemic COVID-19 patient was reported in April 2020.^[10]^ Since then, various studies have reported a very high prevalence of hypocalcemia, ranging from 62.6 to 87.2%.^[11,12]^

Various prediction rules have been reported to predict adverse events of COVID-19, but most have been developed for hospitalized patients rather than for outpatients. Predicting the severity of COVID-19 at the time of hospital admission can help clinicians appropriately triage and plan follow-up care for patients, and enable better management of limited healthcare resources.^[13]^ This study aimed to assess the predictive value of baseline electrolyte imbalances in distinguishing COVID-19 patients who initially present with mild symptoms at admission but return later with a more severe clinical presentation and experience adverse events, including intensive care unit (ICU) admission, mechanical ventilation, or death.

## 2. Materials and Methods

### 2.1. Study design and patients

This retrospective study was conducted at the Health Sciences University, Atatürk Sanatorium Training and Research Hospital, which is a tertiary healthcare hospital in Ankara, Turkey. All consecutive patients with confirmed COVID-19 were evaluated between August and September 2020. This study was reviewed and approved by the institutional clinical research ethics committee (approval number: 2012-KAEK-15/2494). The requirement for written informed consent was waived due to the retrospective nature of the study, and the data were analyzed anonymously.

Adult patients admitted to our hospital with a diagnosis of COVID-19, confirmed by positive real-time reverse-transcriptase polymerase chain reaction testing of nasal or throat swabs, were included. Diseases other than COVID-19 (lung cancer, pneumonia other than SARS-COV2, etc) might affect and mask COVID-19 related changes in serum electrolyte levels. Therefore, we excluded patients who were initially hospitalized for diseases other than COVID-19 to avoid possible biases. Patients who received corticosteroids were excluded from this study. Patients were evaluated for inclusion if they had a serum electrolyte study within 48 hours of hospital admission. The decision to perform biochemical analyses was made clinically and was not influenced by retrospective study design. Patients with no data on COVID-19 outcomes were excluded. After the screening, 286 patients were included in the final analysis.

### 2.2. Data collection

Data were collected from the physical and computerized medical records of our hospital and from the national surveillance system. Data were recorded on a standardized form that was evaluated and approved by the local clinical research ethics committee. Respiratory medicine clinicians who treated COVID-19 patients collected clinical data.

The variables collected included age, sex, symptoms, comorbidities, lymphocyte count, serum calcium, plasma sodium and potassium, lactate dehydrogenase (LDH), ferritin, C-reactive protein (CRP), D-dimer, troponin levels, and estimated glomerular filtration rate (as estimated by the Chronic Kidney Disease Epidemiology Collaboration equation). When blood glucose levels were >180 mg/dL, plasma sodium concentrations were corrected for hyperglycemia.^[14]^ Total serum calcium levels were corrected for albumin levels measured using the same blood test.^[15]^ We excluded all serum electrolyte values measured using a blood gas analyzer.

The peripheral oxygen saturation levels were recorded on admission. Data on clinical outcomes included hospitalization, ICU admission, death within 30 days of diagnosis, and length of hospital stay.

### 2.3. Statistical analysis

The conformity of the data to a normal distribution was evaluated using skewness and kurtosis tests, and histogram plots. Normally distributed continuous variables are presented as mean and standard deviation (SD). Non-normalized variables were presented as medians with ranges or interquartile ranges. Descriptive statistics of the data are presented as counts and percentages for categorical variables. The significance level was set at *P* = .05. Bivariate correlations between variables were assessed using Pearson correlation coefficient (*r*) for normally distributed variables, and Spearman correlation coefficient (*r*_*s*_) for other variables. Categorical variables were compared using the chi-square test and Fisher exact test when the expected cell count was <5. Normally distributed variables were compared using the *t* test for independent samples, and non-normalized variables were compared using the Mann–Whitney *U* test.

Receiver operating characteristic (ROC) curve analysis was performed to evaluate the predictive performance of serum calcium, sodium, and potassium levels on the admission of COVID-19 patients. Logistic regression was performed with variables of total serum calcium concentration as hypocalcemia, serum sodium, and potassium levels, troponin, and D-dimer levels on the likelihood that patients have severe COVID-19 enough to be hospitalized. The patient’s outcome as hospitalized or outpatient after COVID-19 diagnosis was the dependent variable in binary logistic regression. There were no highly correlated variables among the independent variables; therefore, multicollinearity was not present in the model. The logistic regression model was statistically significant, (*χ*^2^ = 148.830, *P* < .001). The explained variation in the dependent variable was 76.2% (Nagelkerke *R*^2^) in our model, and correctly classified 95.1% of cases. Statistical analyses were performed using IBM SPSS Statistics (version 28.0.1.1; IBM Corp., Armonk, NY).

## 3. Results

### 3.1. Patient characteristics

In total, 286 patients with confirmed COVID-19 (139 male and 147 female) were included in the final analysis. The mean age of patients was 49.1 years (±14.5). Triage and treatment of these patients was performed in accordance with the novel coronavirus pneumonia diagnosis and treatment guidelines (April 14, 2020, edition, published by the National Health Commission of Turkey).^[16]^ Chest radiography, complete blood count, and biochemical analyses of serum with standard parameters (including electrolyte levels, inflammatory markers, and thrombosis markers) were performed on the first admission of all patients suspected to have COVID-19.

In accordance with the World Health Organization severity definitions, 45 patients with severe diseases requiring oxygen support, and patients with critical disease with complications were hospitalized. Patients who were not severe enough to be hospitalized were followed up as outpatients by their family physicians at regular periods, and their status was recorded by the national surveillance system for COVID-19. Hospitalized patients were older (60.1 (±11.8)) than outpatients (47.1 (±14.1)) (*P* < .001). There was male predominance in hospitalized patients (57.8%) than outpatients (46.9%), but the difference was not statistically significant (*P* = .18).

The most common preexisting comorbidity was hypertension (11.5%), followed by diabetes mellitus (7.3%), asthma (4.5%), coronary artery disease (4.2%), and chronic obstructive pulmonary disease (2.1%). The number of comorbidities was higher in hospitalized patients than in non-hospitalized patients; however, the difference was statistically insignificant (*P* = .133) predominant symptoms were fever (38.2%), cough (36.8%), fatigue (31.4%), myalgia (28.7 %), and dyspnea (11.8%). As expected, peripheral oxygen saturation was significantly lower in hospitalized patients than in outpatients (*P* < .001). As COVID-19 severity markers, lymphocyte count was significantly lower, and LDH, CRP, ferritin, troponin, and D-dimer levels were significantly higher in hospitalized patients (Table 1).

**Table 1:** Demographic and laboratory parameters of patients at the admission.

	Outpatients (non-severe COVID-19) (n = 241), mean (±SD)	Hospitalized (severe COVID-19) (n = 45), mean (±SD)	*P*
Demographics			
Age (yr)	47.1 (±14.1)	60.1 (±11.8)	<.001
Gender, female, n (%)	128 (53.1%)	19 (42.2%)	.180
Duration of symptoms, d	3.1 (±1.6)	5.0 (±1.9)	.002
Number of comorbidities			.133
0	176 (73.0%)	25 (55.6%)	
1	45 (18.7%)	14 (31.1%)	
2	16 (6.6%)	5 (11.1%)	
3	4 (1.7%)	1 (2.2%)	
Laboratory parameters			
Total serum calcium concentration, mg/dL	9.56 (±0.51)	8.88 (±0.55)	<.001
Plasma sodium concentration, mEq/L	138.9 (±2.3)	135.9 (±4.2)	<.001
Plasma potassium concentration, mEq/L	4.11 (±0.34)	4.05 (±0.51)	.376
Lymphocyte count, x10^3^/µL, median (IQR)	1667 (782–2552)	1090 (520–1660)	<.001
Albumin, g/dL	4.35 (±0.33)	3.66 (±0.48)	<.001
Glucose, mg/dL, median (IQR)	115.4 (82.4–148.4)	133.4 (77.9–188.9)	.078
eGFR, mL/min/1.73m^2^	91.0 (±17.5)	73.6 (±23.5)	<.001
LDH, U/L, median (IQR)	197.9 (143.9–251.9)	380.4 (206.4–554.4)	<.001
CRP, mg/L	10.8 (±17.6)	83.8 (±76.6)	<.001
Ferritin, ng/mL	137.2 (±159.0)	760.7 (±594.8)	<.001
Troponin, ng/L	4.7 (±7.6)	33.7 (±75.6)	<.001
D-dimer, mg/L, median (IQR)	0.32 (0.03–0.61)	0.76 (0.01–1.52)	<.001

COVID-19 = coronavirus disease 2019, CRP = C-reactive protein, eGFR = estimated glomerular filtration rate, IQR = interquartile range, LDH = lactate dehydrogenase, SD = standard deviation.

The duration of hospital stay was 10.0 (±11.4) days. Seven (15.6%) patients were transferred to ICU to escalate care needs. Eleven patients (3.9%) died within the first 30 days of COVID-19 diagnosis. All patients were hospitalized. The case fatality rate was 24.4% among hospitalized patients.

### 3.2. Baseline electrolyte disorders of patients

**Figure 1.**
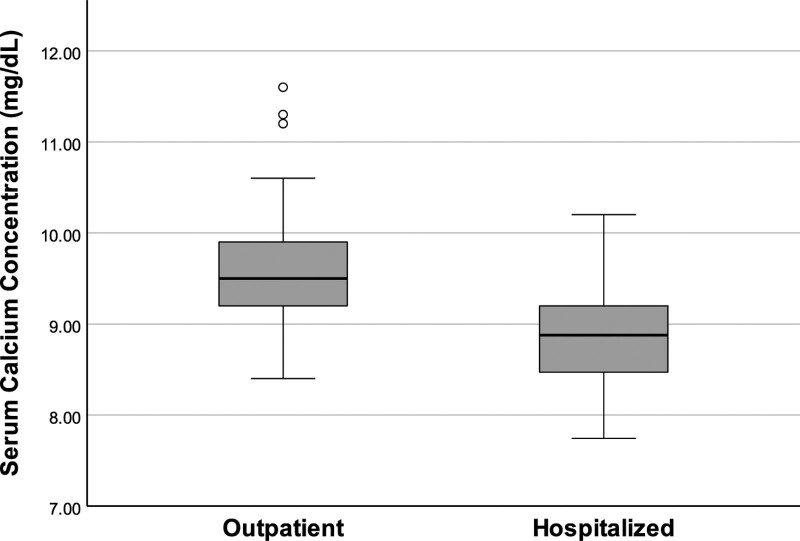
Comparison of baseline serum calcium concentrations of outpatients and hospitalized patients. The mean serum calcium concentration of hospitalized patients was lower than outpatients (*P* < .001).

**Figure 2.**
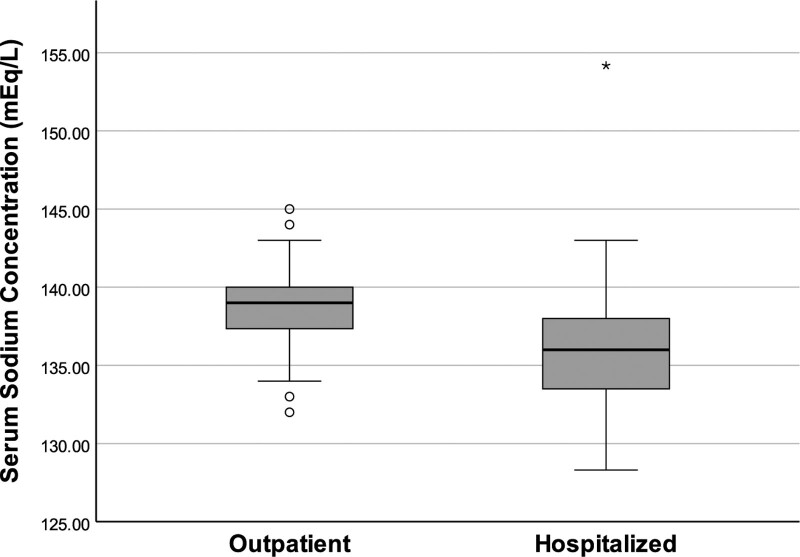
Comparison of baseline serum sodium concentrations of outpatients and hospitalized patients. The mean serum sodium concentration of hospitalized patients was lower than outpatients (*P* < .001).

The mean (±SD) total serum calcium concentration of hospitalized patients (8.88 [±0.55] mg/dL) was significantly lower than that of the outpatients (9.56 [±0.51] mg/dL) (*P* < .001) (Fig. 1). The mean (±SD) serum sodium concentration of hospitalized patients (135.9 [±4.2] mEq/L) was also significantly lower than that of outpatients (138.9 [±2.3] mEq/L) (*P* < .001) (Fig. 2). The mean (±SD) plasma potassium levels of hospitalized patients (4.05 [±0.51] mEq/L) were lower than those of outpatients (4.11 [±0.34] mEq/L), but the difference was not statistically significant (*P* = .376).

Hypercalcemia (>10.6 mg/dL in serum) was found only in 3 (1.0%) outpatients. Hypocalcemia (<8.8 mg/dL in serum) was observed significantly higher in hospitalized patients on admission (46.7%) than outpatients (5.4%) (*P* < .001). Disnatremia was observed more frequently in hospitalized patients (62.2%) than outpatients (12.9%) (*P* < .001). Hypernatremia (>155 mEq/L in serum) and hyponatremia (<135 mEq/L in serum) were observed in 2.2% and 60% of hospitalized patients, respectively. In outpatients, only hyponatremia was observed (12.9%). Hypopotassemia (<3.5 mEq/L) prevalence was significantly higher in hospitalized patients (13.3%) than outpatients (2.5%) (*P* = .001).

### 3.3. Predictive performance of baseline electrolyte concentrations to predict the severe COVID-19 course that required hospitalization and 30-day mortality

Baseline electrolyte concentrations in the blood of the patients were significantly correlated with inflammatory and severity marker levels. Calcium, sodium, and potassium concentrations were positively correlated with lymphocyte count (*r*_*s*_ = 0.297, *P* < .001; *r*_*s*_ = 0.217, *P* < .001; and *r*_*s*_ = 0.121, *P* = .043, respectively) and negatively correlated with troponin level (*r* = −0.133, *P* = .028; *R* = 0.167, *P* = .005; and *r* = −0.138, *P* = .021, respectively). Calcium and sodium concentrations (but not potassium) were negatively correlated with baseline LDH level (*r*_*s*_ = −0.325, *P* < .001; *r*_*s*_ = −0.325, *P* < .001, respectively), baseline CRP value (*r* = −0.262, *P* < .001; *r* = −0.314, *P* < .001, respectively), baseline ferritin value (*r* = −0.314, *P* < .001; *r*_*s*_ = −0.288, *P* < .001, respectively), and baseline D-dimer level (*r*_*s*_ = 0.157, *P* = .01; *r*_*s*_ = −0.196, *P* = .001).

**Figure 3.**
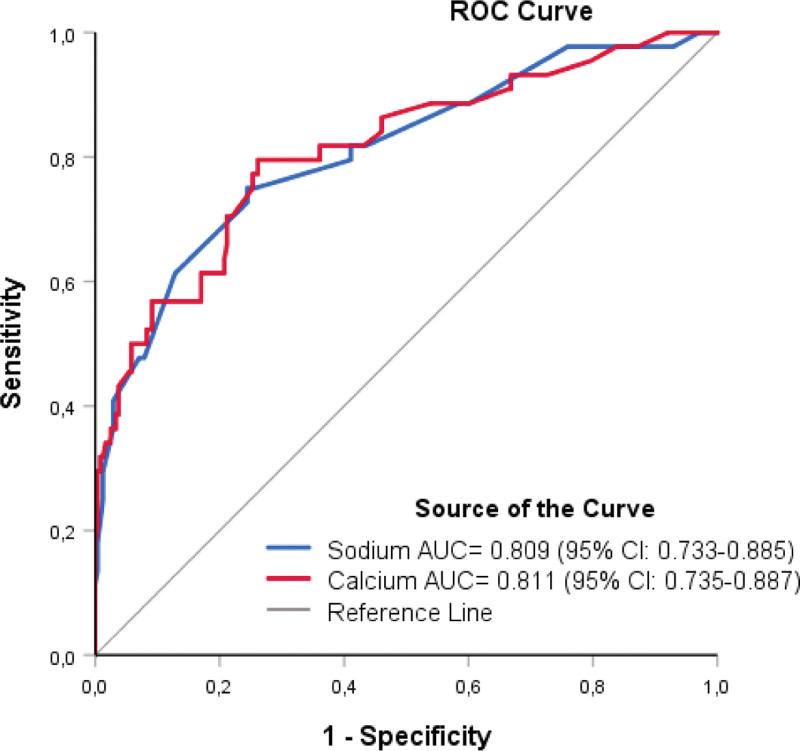
Receiver operating characteristic (ROC) curve analyses of the predictive performance of baseline serum calcium and sodium concentrations of patients to forecast severe COVID-19 enough to be hospitalized. AUC = area under the ROC curve, CI = confidence interval, COVID-19 = coronavirus disease 2019.

We evaluated the prognostic performance of the baseline serum total calcium, sodium, and potassium concentrations using ROC analysis to predict whether patients with severe COVID-19 were enough to be hospitalized. Hypocalcemia and hyponatremia effectively predicted hospitalization, with area under the ROC curve (AUC) of calcium and sodium levels of 0.811 (95% confidence interval [CI]: 0.735–0.887; *P* < .001), and 0.809 (95% CI: 0.733–0.885; *P* < .001) (Fig. 3). The area under the curve of potassium level (0.570) was not statistically significant (*P* = .142).

**Figure 4.**
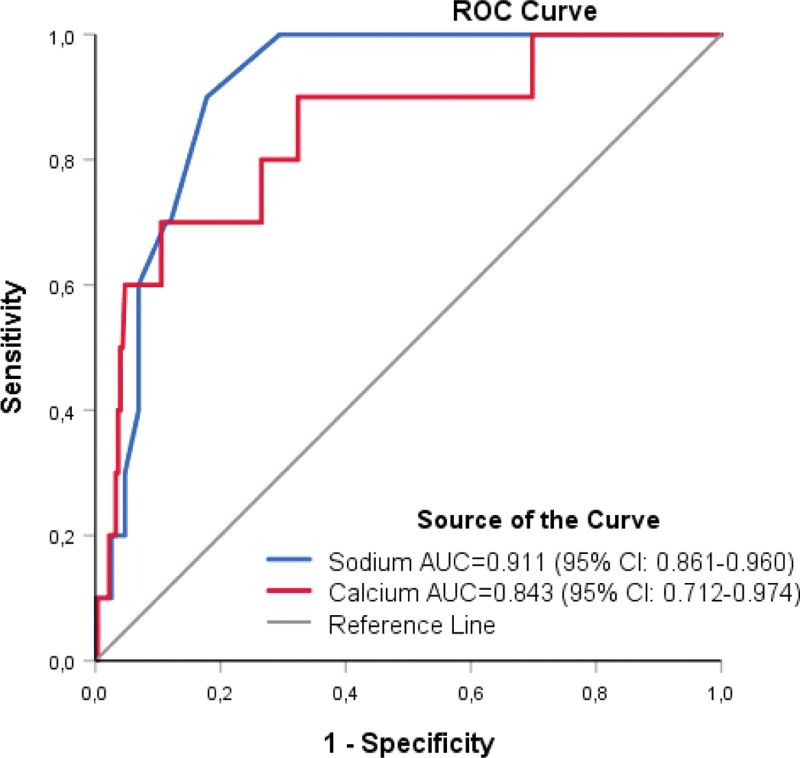
Receiver operating characteristic (ROC) curve analyses of the predictive performance of baseline serum calcium and sodium concentrations of patients to forecast 30-day mortality of COVID-19. AUC = area under the ROC curve, CI = confidence interval, COVID-19 = coronavirus disease 2019.

The case fatality rate was significantly higher (20.5%) in hypocalcemic patients than in normocalcemia patients (1.6%) (*P* < .001). The AUC of baseline serum calcium levels for predicting 30-day mortality was 0.843 (95% CI: 0.712–0.974; *P* < .001). The case fatality rate was significantly higher also (15.5%) in hyponatremic patients than normonatremic patients (0.9%) (*P* < .001). The AUC of baseline serum sodium level for predicting 30-day mortality was 0.911 (95% CI: 0.861–0.960; *P* < .001) (Fig. 4). The difference in the case of fatality rates between hypopotassemic (18%) and normopotassemic patients (3.3%) was not statistically significant (*P* = .061).

Multivariable logistic regression was performed to ascertain the effects of serum calcium, sodium, and potassium levels with troponin and D-dimer levels on the likelihood that patients had severe COVID-19 enough to be hospitalized. The logistic regression model was statistically significant (*χ*^2^ = 148.830, *P* < .001). In this model, the total serum calcium concentration as hypocalcemia (*β* = 2.019, *P* = .01), troponin (*β*  = 0.029, *P* = .002), and D-dimer levels (*β* = 1.503, *P* = .035) at admission were predictive of hospitalization. COVID-19 patients who were hypocalcemic at admission were 7.53 times (95% CI: 1.63–34.71) more likely to exhibit severe disease enough to be hospitalized than normocalcemic patients.

## 4. Discussion

Electrolyte disorders are common in patients with COVID-19, especially hypokalemia, hyponatremia, and hypocalcemia, which are frequently observed in severe COVID-19.^[17–19]^ Electrolyte disorders have also been reported in Ebola and SARS-like viral infections during previous pandemics.^[20,21]^ Hyponatremia is reported as 2.3 to 44%, hypernatremia 1.1 to 4.4%, hypokalemia 10.2 to 39%, hyperkalemia 0.8 to 13%, and albumin-corrected hypercalcemia 0.7 to 7.5% in an unselected population in the emergency department.^[22]^ Disnatremia was observed more frequently in COVID-19 patients than in other patients.^[23]^

Abnormalities in serum sodium, calcium, and potassium levels at admission were found at 20.6%, 14%, and 4.2%, respectively in our study. While hyponatremia was the most prevalent electrolyte disorder in this study and observed in 60% of hospitalized patients, hypernatremia was observed in only 2.2% of this group. The prevalence was lower (12.9%) in outpatients. According to disease severity, the prevalence of disease, hyponatremia has been reported to range from 9.9 to 63.6% in COVID-19 patients.^[24–26]^ The water and sodium balance is regulated by water intake pathways (osmoregulation) and antidiuretic hormone, renin-angiotensin-aldosterone system, and natriuretic hormone pathway (volume regulation). The clinical appearance of diminished regulation includes hypernatremia (too little water), hyponatremia (too much water), volume expansion (too much sodium), volume depletion (too little sodium), and polyuria.^[25]^

A small proportion of patients were admitted with symptoms of diarrhea (4.9%), nausea, and vomiting (1.1%) in this study. Hypovolemic hyponatremia caused by gastrointestinal electrolyte loss can contribute to observed hyponatremia in these patients. In this study, the prevalence of hyponatremia was significantly higher in hospitalized patients than that in outpatients. The hospitalized patients were older and had more comorbidities. The glomerular filtration rate levels were significantly lower in hospitalized patients. Decreased ability of the kidneys to regulate electrolytes in COVID-19 patients and renal function deficiency in older subjects can contribute to the observed higher prevalence of hyponatremia in hospitalized patients. COVID-19-associated syndrome of inappropriate antidiuresis resulting from inflammatory cytokine release, intravascular volume depletion, and positive-pressure ventilation has been reported.^[27,28]^ In this study, the serum sodium concentration was found to correlate with inflammatory cytokine release markers.

Hypocalcemia has also been frequently reported as an electrolyte abnormality in COVID-19 patients.^[18]^ In this study, hypocalcemia on admission was observed in 46.7% of patients who were hospitalized later for severe COVID-19. This is similar to the range of 44.8 to 74.7% reported for severe forms of COVID-19 in literature.^[19,29]^ The baseline hypocalcemia prevalence was significantly lower in patients who were followed up as outpatients owing to the lower disease severity in this study. Several studies have reported that hypocalcemia correlates with inflammation, thrombosis markers, disease severity, and mortality in COVID-19 patients.^[11,12]^ Serum calcium concentration was correlated with inflammation and thrombosis markers, which is in agreement with the results of these studies. Hypocalcemia may also be associated with imbalanced vitamin D and parathormone levels in the acute phase of COVID-19.^[30]^

Hypopotassemia is also a prevailing electrolyte disorder in COVID-19 patients due to the interaction of SARS-CoV-2 with the renin-angiotensin-aldosterone system. Hypopotassemia has been reported in up to 40% of hospitalized patients.^[31]^ The prevalence was found at 13.3% in hospitalized patients and 2.5% in outpatients in this study. Hypopotassemia is particularly important because it may increase the prevalence of potentially fatal arrhythmias in COVID-19 patients.^[32]^

Abnormal electrolyte levels during hospitalization are associated with clinical severity and prognosis of patients with COVID-19.^[33–35]^ Although it has been well-established for hospitalized patients, there is a paucity of data on patients admitted with mild symptoms during the early stages of COVID-19. When we evaluated the prognostic performance of baseline serum electrolyte concentrations of patients admitted to our hospital with newly diagnosed COVID-19, hypocalcemia and hyponatremia effectively predicted hospitalization and 30-day mortality in the ROC curve analyses. Hypocalcemia, detected at the first evaluation upon hospital admission, was identified in multivariate logistic regression analysis as an independent risk factor associated with the risk of hospitalization.

Although most COVID-19 patients have developed only mild (40%) or moderate (40%) disease, 15% develop a severe disease that requires oxygen support, and 5% may rapidly and unpredictably progress to a critical disease requiring intensive care treatment.^[36]^ Distinguishing critical laboratory biomarkers for disease severity at an early stage could help to monitor and prevent disease progression to a severe form. Risk stratification at the time of hospital presentation is important to allow the early identification of patients who are most likely to have poor outcomes and effective allocation of health resources, especially in economically developing countries.^[37,38]^ As a widely available and affordable laboratory test and marker of the inflammatory response, the measurement of serum electrolyte concentrations can reliably assist in the risk stratification of patients with COVID-19 at admission and facilitate timely appropriate care.

### 4.1. Study limitations

Owing to logistical limitations at the beginning of the COVID-19 pandemic, urine sodium, chloride concentrations, and urine volumes were not obtained in the emergency and COVID-19 wards, which limited the classification of patients with hyponatremia. The retrospective study design at a single medical center is also a potential limitation for external validation. We were also unable to study the longitudinal effect of COVID-19 on electrolyte balance due to the short follow-up period.

## 5. Conclusion

Our study demonstrates that hypocalcemia and hyponatremia at hospital admission effectively predict disease progression toward hospitalization and 30-day mortality in patients with COVID-19. Clinicians should closely follow up or reevaluate COVID-19 patients with baseline electrolyte disorders.

## Acknowledgments

The authors thank Mrs İdil Berktaş for designing the figures.

## Author contributions

**Conceptualization:** Nevin Taci Hoca, Bahadir M Berktaş.

**Data curation:** Bahadir M Berktaş.

**Formal analysis:** Bahadir M Berktaş.

**Investigation:** Nevin Taci Hoca, Bahadir M Berktaş.

**Methodology:** Nevin Taci Hoca, Bahadir M Berktaş.

**Project administration:** Nevin Taci Hoca, Bahadir M Berktaş.

**Resources:** Nevin Taci Hoca, Bahadir M Berktaş.

**Software:** Bahadir M Berktaş.

**Supervision:** Bahadir M Berktaş.

**Validation:** Nevin Taci Hoca, Bahadir M Berktaş.

**Visualization:** Nevin Taci Hoca, Bahadir M Berktaş.

**Writing – original draft:** Nevin Taci Hoca, Bahadir M Berktaş.

**Writing – review & editing:** Nevin Taci Hoca, Bahadir M Berktaş.
